# Exploring Predictive and Prognostic Biomarkers in Colorectal Cancer: A Comprehensive Review

**DOI:** 10.3390/cancers16162796

**Published:** 2024-08-08

**Authors:** Karam Ashouri, Alexandra Wong, Pooja Mittal, Lesly Torres-Gonzalez, Jae Ho Lo, Shivani Soni, Sandra Algaze, Taline Khoukaz, Wu Zhang, Yan Yang, Joshua Millstein, Heinz-Josef Lenz, Francesca Battaglin

**Affiliations:** 1 Division of Medical Oncology, Norris Comprehensive Cancer Center, Keck School of Medicine, University of Southern California, Los Angeles, CA 90089, USA; karam.ashouri@med.usc.edu (K.A.); alexandra.wong@med.usc.edu (A.W.);; 2 Department of Population and Public Health Sciences, Norris Comprehensive Cancer Center, Keck School of Medicine, University of Southern California, Los Angeles, CA 90089, USA

**Keywords:** colorectal cancer, gene expression profiling, molecular targeted therapy, tumor biomarkers

## Abstract

**Simple Summary:**

Colorectal cancer is a major health concern globally, and finding ways to improve treatment outcomes is crucial. This review explores the role of biomarkers—biological indicators that can predict how a patient will respond to treatment or indicate the likely course of the disease—in managing colorectal cancer. By examining both well-established and emerging biomarkers, we hope to provide a clearer understanding of how these markers can guide personalized treatment plans. The findings from this research could help doctors make more informed decisions, ultimately improving patient care and outcomes in colorectal cancer.

**Abstract:**

Colorectal cancer (CRC) remains the second leading cause of cancer-related mortality worldwide. While immune checkpoint inhibitors have significantly improved patient outcomes, their effectiveness is mostly limited to tumors with microsatellite instability (MSI-H/dMMR) or an increased tumor mutational burden, which comprise 10% of cases. Advancing personalized medicine in CRC hinges on identifying predictive biomarkers to guide treatment decisions. This comprehensive review examines established tissue markers such as KRAS and HER2, highlighting their roles in resistance to anti-EGFR agents and discussing advances in targeted therapies for these markers. Additionally, this review summarizes encouraging data on promising therapeutic targets and highlights the clinical utility of liquid biopsies. By synthesizing current evidence and identifying knowledge gaps, this review provides clinicians and researchers with a contemporary understanding of the biomarker landscape in CRC. Finally, the review examines future directions and challenges in translating promising biomarkers into clinical practice, with the goal of enhancing personalized medicine approaches for colorectal cancer patients.

## 1. Introduction

Colorectal cancer (CRC) is the third most common cancer and the second leading cause of cancer-related deaths worldwide [1]. Approximately 20% of patients have metastatic disease upon presentation, and the 5-year survival rate for these patients remains less than 20%, highlighting the need to expand the therapeutic armamentarium in this setting [2].

Over the last two decades, the treatment landscape for metastatic CRC (mCRC) has significantly evolved. The standard of treatment was 5-fluorouracil (5-FU) combined with leucovorin until it was found that combining 5-FU with oxaliplatin or irinotecan improved median overall survival (OS) to 20 months [3]. The advent of immunotherapy has also led to substantial therapeutic benefits, with a 4-year OS > 70% in mCRC positive for microsatellite instability or mismatch repair deficiency (MSI-H/dMMR) [4]. However, this biomarker applies to 4% of all mCRC cases, leaving most patients reliant on traditional chemotherapy and incremental gains from new biologic agents. 

The identification of predictive and prognostic biomarkers with immunohistochemistry (IHC) or next-generation sequencing (NGS) is crucial for advancing personalized medicine in CRC. Predictive biomarkers help determine the likelihood of the response to specific therapies, while prognostic biomarkers provide information on the overall disease outcome, regardless of treatment. Notably, CRC primary tumors and metastatic sites harbor a high concordance (>90%) in their mutational profile for actionable genes [5,6]. Integrating these genetic markers in the four distinct consensus molecular subtypes (CMSs) has enriched our understanding of the clinical and biological characteristics of CRC [7].

Despite the progress in molecular diagnostics, challenges remain in translating these biomarkers into clinical practice. The heterogeneity of CRC, both morphologically and molecularly, complicates the identification of universally applicable biomarkers. The clinical relevance of many emerging biomarkers is still under investigation, necessitating further research and validation in larger patient cohorts. This review aims to provide a comprehensive overview of key established, promising, and potential biomarkers in CRC.

## 2. Established Biomarkers

### 2.1. RAS Status

Proteins of the RAS family are GTPases involved in the RAS/RAF/MEK/ERK pathway, playing a crucial role in cell division and proliferation. RAS mutations are prevalent across various cancers, making them one of the most common oncogenes. In CRC, RAS mutations are found in approximately 55% of cases, with *KRAS* mutations occurring in about 50%, *NRAS* in 4%, and *HRAS* in less than 1% [8].

**Figure 1:**
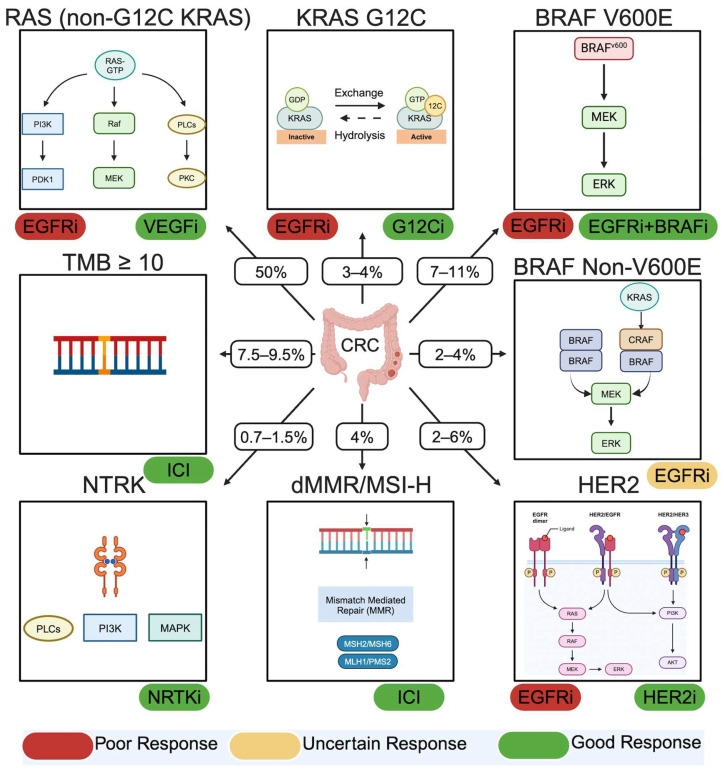
Current predictive biomarkers for metastatic colorectal cancer (CRC). EGFRi = EGFR inhibition; VEGFi = VEGF inhibition; G12Ci = KRAS G12C inhibition; BRAFi = BRAF inhibition; ICI = Immune checkpoint inhibition; HER2i = HER2 inhibition; CRC = colorectal cancer. (Created in Biorender.com).

Most *KRAS* mutations involve glycine substitutions at positions 12 or 13, leading to constitutive activation of the GTPase side chain. The most common *KRAS* mutations in CRC are *KRAS* G12D (36%), *KRAS* G12V (21.8%), and *KRAS* G13D (18.8%), while *KRAS* G12C mutations are found in 3–4% of CRC cases (Figure 1) [9,10]. *KRAS* mutations are more prevalent in left-sided tumors than right-sided tumors and similarly more common in microsatellite stable (MSS) CRC relative to MSI-H/dMMR CRC. In frontline chemotherapy–treated mCRC, the *KRAS* wildtype status conferred an improved OS relative to mutated tumors (20.9 months vs. 16.9 months, HR = 0.71, 95% CI 0.57, 0.88, HORIZON II, NCT00399035) [11]. The prognostic utility of KRAS mutations extends to both young-onset and late-onset CRC, revealing worse cause-specific survival relative to KRAS wildtype tumors in both age groups [12]. Among specific *KRAS* mutations, G12C demonstrated the worst prognosis relative to wildtype tumors (4.3 months vs. 23.3 months, *p* < 0.001) [13]. 

RAS mutations result in constitutive activation downstream of EGFR blockade, rendering epidermal growth factor receptor (EGFR) inhibitors ineffective. Multiple studies, including the phase 3 CRYSTAL and PRIME trials, have demonstrated that *KRAS*/*NRAS* mutations predict a poor response to combination chemotherapy with EGFR inhibitors [14,15]. Consequently, testing for *KRAS*/*NRAS* mutations is recommended by the National Comprehensive Cancer Network (NCCN) for all patients with mCRC, because those with these mutations should not receive panitumumab or cetuximab in combination with chemotherapy or as monotherapy [16]. Within 12 months of treatment, most CRCs acquire resistance to anti-EGFR therapies, frequently (~50%) due to secondary KRAS alterations [17,18]. This underscores the importance of checking for these mutations during anti-EGFR therapy.

When selecting biologic agents (bevacizumab, cetuximab, and panitumumab) to pair with frontline chemotherapy in mCRC, several studies highlight the superiority of anti-EGFR therapies (cetuximab and panitumumab) in RAS wildtype/left-sided tumors (Figure 1). The PARADIGM Study demonstrated improved OS with panitumumab versus bevacizumab in combination with chemotherapy for first-line treatment in RAS wildtype patients (37.9 months vs. 34.3 months), with a more pronounced effect in left-sided tumors [19]. Updated results from the RAS wildtype cohort in the FIRE-3 trial indicated improved OS with the FOLFIRI plus cetuximab group compared to the FOLFIRI plus bevacizumab group (33.1 months vs. 25.0 months; HR = 0.70; *p* = 0.0059) [20]. The cetuximab group also demonstrated a more frequent early tumor shrinkage and a greater median depth of response [20]. 

Recent advances have led to the development of oral *KRAS* G12C small molecule inhibitors. Initial studies of sotorasib and adagrasib monotherapy showed modest effects in chemo-refractory mCRC, and combining them with EGFR inhibitors appears to enhance their efficacy and overcome resistance mechanisms [21]. The KRYSTAL-1 trial, a phase I/II study examining adagrasib with or without cetuximab, showed a numerically higher median duration of response and response rates with the addition of cetuximab [22] (NCT03785249). Soon after, adagrasib was awarded accelerated Food and Drug Administration (FDA) approval as a second-line agent in combination with cetuximab for *KRAS* G12C–mutated CRC. The phase 3 CodeBreak 300 trial evaluates sotorasib in chemo-refractory mCRC at two doses (240 mg and 960 mg) in combination with panitumumab compared to a standard of care (SOC) group (Lonsurf or regorafenib) [23]. Early data demonstrate superior progression free survival (PFS) in both sotorasib groups relative to SOC (960 mg: 5.6 months vs. 240 mg: 3.9 months vs. SOC: 2.2 months) (NCT05198934) [23]. Another *KRAS* G12C inhibitor, divarasib, is being studied in a Phase 1 trial as monotherapy and in combination with cetuximab (NCT04449874) (Table 1). Additional phase 3 studies will examine adagrasib combined with cetuximab compared to chemotherapy in the second-line setting (NCT04793958) and sotorasib in combination with panitumumab and chemotherapy in treatment-naive patients (NCT06252649). Preclinical data suggest that SHP2-inhibitors augment *KRAS* G12C inhibitors by improving engagement and downregulating resistance mechanisms, leading to clinical trials evaluating this combination (NCT04330664, NCT04916236, NCT05288205, NCT04699188, NCT04449874) (Table 1) [24]. In preclinical CRC models, *KRAS* mutations increased the expression of amino acid transporters via the hippo pathway effector YAP1, resulting in mTOR activation and subsequent CRC cell proliferation [25]. Mechanistic studies in lung cancer indicate that the hippo pathway is implicated in *KRAS* G12C resistance, highlighting its therapeutic potential in augmenting KRAS inhibitors [26]. G12D is the most prevalent *KRAS* alteration in CRC, with several small molecular inhibitors (MRTX1133 and RMC-9805) undergoing early phase evaluation (NCT05737706, NCT06040541) (Table 1). MRTX1133 (G12D inhibitor) has shown synergism alongside 5-FU in preclinical models, with similar effects in *KRAS* G12V–mutated cell lines [27]. Alternative strategies targeting specific *KRAS* mutations utilize T cell receptors (TCRs), which recognize intracellular antigens presented by human leukocyte antigen (HLA) molecules to trigger an immune response [28]. Several clinical trials utilize TCRs in *KRAS* G12V (NCT06105021, NCT06043713) and G12D (NCT03948763) CRC (Table 1). In addition to the mutation selective inhibitors, clinical trials are evaluating pan-KRAS inhibitors (NCT04975265) (Table 1). RMC-6236 is a pan-RAS inhibitor that targets the full range of RAS alterations (*KRAS*, *HRAS*, *NRAS*) with activity against mutant and wildtype RAS variants and is undergoing Phase 1 studies (NCT05379985) (Table 1) [29].

### 2.2. BRAF Mutations

The RAF family of serine/threonine kinases are components of the RAS/RAF/MAPK/ERK signaling pathway and contain the following three members: ARAF, BRAF, and CRAF [30]. Under physiological conditions, RAF proteins exist as monomers within the cytosol until they are recruited to the membrane, bind activated RAS, and form active homo- or hetero-dimers [31]. Once activated, RAF dimers go on to activate MEK1/2, subsequently activating the ERK1/2 transcription complex, which regulates cell proliferation, differentiation, survival, senescence, and apoptosis [30,31,32]. The most common *BRAF* mutation results in valine substitution for glutamic acid at amino acid 600 (*BRAF^V600E^*), stimulating the MEK/ERK pathway in tumor cells (Figure 1) [33,34]. Clinical and pathological phenotypes associated with *BRAF^V600E^*-mutated CRC include proximal tumor location, aggressive growth, unfavorable metastasizing patterns, EGFR blockade resistance, and reduced overall survival (Figure 1) [35]. Pooled analysis from three mCRC clinical trials (COIN, FOCUS, PICCOLO) noted an increased frequency of MSI-H/dMMR with BRAF mutations compared to the BRAF wildtype (12.6% vs. 3.0%, *p* < 0.001) [36]. When combining the two frontline trials (COIN, FOCUS), BRAF mutant tumors suffered from worse OS (10.8 months vs. 16.4 months, HR  =  1.49, *p*  <  0.001) and remained significant when adjusting for confounders [36]. The negative prognostic impact of BRAF mutations has been confirmed in several other studies [37,38,39,40]. Non-V600E *BRAF* mutations occur less frequently in mCRC (2.2% vs. 7.8%) but offer better prognoses than those with the V600E mutation (*p* < 0.001) (Figure 1) [41]. Several retrospective studies and meta-analyses identified BRAF V600E as a negative predictor for a response to anti-EGFR therapy [42,43,44]. However, the predictive value of BRAF non-V600E mutations remains unclear (Figure 1) [45,46]. 

Therapies that target mutated *BRAF* include the SOC in melanoma, non-small cell lung cancer, and some thyroid malignancies. However, monotherapy with vemurafenib or dabrafenib is ineffective in V600E mCRC due to alternative MAPK activation mechanisms maintaining elevated MEK activity [35,47]. Co-inhibition of *BRAF* and the alternative activation mechanisms targeting EGFR or MEK have demonstrated successful reduction in MAPK signaling activity and improved patient treatment response [48,49,50]. Compared to chemotherapy control (FOLFIRI or irinotecan + cetuximab) in pretreated *BRAF^V600E^* mCRC, both doublet (encorafenib + cetuximab) and triplet therapy (encorafenib + binimetinib + cetuximab) demonstrated superior OS (5.9 months vs. 9.3 months vs. 9.3 months, respectively) and PFS (1.5 months vs. 4.3 months vs. 4.5 months, respectively) in the phase III BEACON trial [51]. Given that adverse events were more frequent with triplet therapy but had similar efficacy, the encorafenib plus cetuximab combination is an approved second-line regimen in BRAF^V600E^ mCRC, with ongoing trials evaluating it in the first-line setting (Figure 1) (phase III BREAKWATER trial NCT04607421). 

Notably, preclinical models in microsatellite stable (MSS) CRC demonstrate that combined BRAF/EGFR inhibition induces a transient MSI-H/dMMR phenotype [52]. Encorafenib, cetuximab, and nivolumab combination therapy in MSS/*BRAF^V600E^* recently demonstrated encouraging PFS (7.4 months) and OS (15.1 months) with a randomized phase II trial underway [53] (NCT05308446).

### 2.3. HER2

HER2, encoded by *ERBB2*, is an EGFR tyrosine kinase family member. Its overexpression and/or amplification have been reported in various solid tumors, particularly breast cancer, where HER2-targeted therapies have been highly successful. The NCCN Guidelines now recommend HER2 testing for all mCRC patients, present in 2–6% of cases given therapeutic implications with anti-EGFR and HER2 directed therapy (Figure 1). HER2 is measured by IHC, fluorescence in situ hybridization (FISH), or NGS. HER2 overexpression is indicated by an IHC score of 3+, while an IHC score of 2+ is equivocal and should prompt FISH testing. A FISH ratio ≥2 confirms HER2 amplification [54]. HER2 amplification is more common in left-sided tumors and is more prevalent in RAS/*BRAF* wildtype tumors [55,56]. Studies have shown that HER2 overexpression may confer resistance to EGFR inhibition (Figure 1) [55,57,58]. On the other hand, a recent analysis from the CALGB/SWOG 80405 trial found that high HER2 expressing mCRC (tumor gene expression above the median) had a longer PFS and OS than tumors with lower *HER2* expression [59]. In patients with lower HER2 expression, treatment with cetuximab was associated with a worse PFS and OS compared to bevacizumab. The poor prognostic implication of decreased HER2 expression was confirmed in RAS wildtype mCRC based on the MONSTAR-SCREEN-2 cohort [60]. These findings suggest that HER2 expression is prognostic and may contribute to selecting anti-EGFR versus anti-VEGF treatment in the first-line metastatic setting [59]. However, other studies have reported mixed findings regarding the prognostic role of HER2, and further prospective validation is needed [61].

HER2 is an established therapeutic target in CRC. In the second-line setting for mCRC, RAS/*BRAF* wildtype patients with HER2 overexpression/amplification who have not received prior HER2 inhibitors can be treated with trastuzumab in combination with pertuzumab, lapatinib, or tucatinib. The HERACLES trial, a phase 2 study of heavily pre-treated patients with the *KRAS* wildtype, HER2+ mCRC, demonstrated the efficacy of dual HER2 blockade using trastuzumab and lapatinib, with an objective response rate (ORR) of 30% [56]. The MyPathway basket study of trastuzumab and pertuzumab in treatment-refractory CRC reported an ORR of 38% [62]. The combination of trastuzumab and tucatinib was the first HER2-directed treatment approved by the FDA for chemo-refractory mCRC, based on the MOUNTAINEER trial (ORR 38%) [63]. The ongoing phase 3 trial, MOUNTAINEER-03 (NCT05253651), is investigating tucatinib and trastuzumab in combination with chemotherapy in the first-line setting compared to standard care in HER2+ patients. In the DESTINY-CRC01 phase 2 trial of patients with HER2+ mCRC who had received at least two prior lines of therapy, fam-trastuzumab deruxtecan was associated with an ORR of 45% [64]. Notably, fam-trastuzumab deruxtecan is an option for patients who received prior HER2-directed therapy; however, the cohort included only patients with HER2 IHC 3+, and patients must be monitored closely for the potential serious side effect of interstitial lung disease [64]. Future studies will help elucidate ideal sequencing strategies for targeted therapy in this group of patients.

### 2.4. MSI-H

Microsatellites are short tandem repeated sequences spread throughout DNA. Microsatellite instability is caused by defects in the mismatch repair system, most commonly due to somatic mutations of *MLH1*, *MSH2*, *MSH6*, *PMS2*, and *EPCAM*, which lead to defects during DNA replication [65,66]. The prevalence of MSI-H/dMMR decreases with the advancing CRC stage as follows: stage II (20%), then stage III (12%), and stage IV (4%) (Figure 1) [67]. Knowledge of the MSI-H/dMMR status is critical in managing CRC for several reasons. Patients with MSI-H/dMMR tumors must be screened for germline mutations in the mismatch repair genes, which leads to Lynch Syndrome, altering the treatment of patients and relatives [68]. With the introduction of immune checkpoint inhibitors (ICI), MSI-H/dMMR status has offered profound survival benefits and cures for some CRCs [69]. The high neoantigen load of MSI-H/dMMR cancer elicits a strong antitumor immune response, further augmented with ICIs (Figure 1) [70]. Additionally, MSI-H/dMMR CRCs have a favorable tumor microenvironment (TME) with increased tumor-infiltrating lymphocytes and decreased immunosuppressive cells like regulatory T cells (Tregs) and myeloid-derived suppressor cells (MDSCs) [71,72]. ICIs further enhance the activity of these preexisting tumor infiltrating lymphocytes (TILs) to target the tumor. 

The phase II KEYNOTE-016 trial was the first to demonstrate superior ORR (40%) in MSI-H/dMMR mCRC relative to MSS (0%) with pembrolizumab [73]. Pembrolizumab’s superior antitumor activity was confirmed in the KEYNOTE-164 trial, demonstrating a median PFS and OS of 4.1 and 47.0 months, respectively, in pretreated CRC (chemotherapy arm PFS: 2.3 months, OS: 31.4 months) [69,74]. The 50% increase in OS relative to the SOC and 30% PFS plateau at 5 years, all with fewer adverse events in the experimental arm, were unheard of in previous CRC trials. A similar efficacy was demonstrated with nivolumab (phase II Checkmate 142 trial), awarding both agents FDA approval for second-line use in 2017 [75]. Phase III KEYNOTE-177 demonstrated that first-line pembrolizumab (200 mg q3w for up to 2 years) was superior to standard chemotherapy (mFOLFOX6 or FOLFIRI q2w  ±  bevacizumab or cetuximab) in MSI-H/dMMR mCRCs, confirming a longer median PFS (16.5 months vs. 8.2 months, HR 0.60, *p*  =  0.0002) [76]. The effect of nivolumab was augmented with the addition of anti-CTLA4 (ipilimumab) therapy (24-month PFS: 74% and OS: 79%) [77]. Phase III Checkmate-8HW confirmed the superior PFS of nivolumab plus ipilimumab compared to chemotherapy (not reached (NR) vs. 5.9 months; HR 0.21, *p* < 0.0001) after 31.5 months of median follow-up in the first-line setting [4].

Despite the clinical benefit, nearly 30% of MSI-H/dMMR tumors are resistant to ICI [78]. Preclinical data indicate that inhibition of VEGF may promote an immune permissive TME [79,80]. Following encouraging safety and efficacy data, atezolizumab plus bevacizumab plus FOLFOX is being evaluated as a first-line regimen in MSI-H/dMMR mCRC (NCT02997228) [81]. Additional studies combine VEGF inhibitors with pembrolizumab or nivolumab as second-line agents (NCT05035381).

### 2.5. Tumor Mutational Burden (TMB)

Tumor mutational burden (TMB) is an established biomarker for predicting the efficacy and response to ICIs in multiple cancer types, including CRC, independent of the MSI-H/dMMR status [82]. TMB is an independent biomarker, and studies have shown TMB to be elevated in MSI-H/dMMR tumors. A high TMB (TMB > 37.4 mutations/megabase [Mb]) stratified patients with a better response to ICIs in an MSI-H/dMMR mCRC cohort [82]. 

TMB can be expressed as the number of acquired/somatic mutations (coding errors, base substitutions, and insertions/deletions) per Mb of the sequenced DNA. NGS and whole exome sequencing (WES) of the tumor genome are used to measure TMB, and examples of diagnostic NGS panels include MSK-IMPACT (Memorial Sloan Kettering) and TSO500 (Illumina) [83,84]. A high TMB could potentially translate into higher loads of neoantigens, stimulating the immune system to recognize and attack tumor cells and thus increasing sensitivity to immunotherapy [85]. Although only a few somatic mutations may give rise to neoantigens, even fewer of these are processed by major histocompatibility complex (MHC) molecules and recognized by T cells as immunogenic. Still, TMB gives a useful estimation of the tumor neoantigen loads [86]. A recent study by Wang et al. (2022) reported a high TMB as an indicator of better prognosis in patients with *KRAS*-mutated CRC. The study revealed that among CRC patients with *KRAS* mutations, those with higher TMB (TMB > 10 mutations per Mb) had a longer OS compared to the ones with lower TMB (TMB ≤ 10 mutations per Mb) [84].

The US FDA approved the use of pembrolizumab for the treatment of cancer patients with TMB of >10 mutations per Mb in 2020 (Figure 1) [87]. However, the variation in intratumoral heterogeneity is one of the factors to be considered while characterizing TMB across different tumor types. Cancers such as melanoma, non-small cell lung cancer (NSCLC), CRC, and other squamous carcinomas exhibit a high TMB, while pediatric tumors and leukemias show the lowest levels of TMB [86]. Due to variation in TMB by cancer type, more studies are warranted to develop TMB into a robust predictive marker of ICI efficacy.

### 2.6. NTRK Fusions

The neurotrophic tyrosine receptor kinase (NTRK) genes include *NTRK1*, *NTRK2,* and *NTRK3,* which encode TRKA, TRKB, and TRKC, respectively. Binding with their respective ligands leads to the transcription of genes involved in neuronal cell differentiation and survival mediated by the MAPK, PI3K, and PKC signaling pathways [88]. The alterations in the NTRK genes have been implicated in the induction of carcinogenesis in both neuronal and non-neuronal cells. These alterations could be in the form of gene mutations, fusions, and deletions. The NTRK fusions have been reported in several cancers in both adult and pediatric populations, with an approximately 80% frequency in rare cancers (including mammary secretory carcinoma and congenital infantile fibrosarcoma). The frequency of NRTK fusion is comparably less (commonly <5%) in common cancers, with the frequency being 0.7–1.5% in CRC (Figure 1) [89]. 

A recent report by Wang et al. (2022) documented that frequent coalterations in *APC* and *TP53* genes were found with NTRK fusions, while alterations in RAS/*BRAF* were mutually exclusive. NTRK fusion-positive tumors showed a high TMB, MSI-H/dMMR, and enrichment in *POLE*/*POLD1* mutations compared to molecularly unstratified CRC tumors. Typically, the NTRK fusion proteins contain the kinase domain of NTRK juxtaposed with a different gene. In several cases, this leads to the constitutive activation of downstream signaling pathways such as MAPK and PI3K. NTRK fusions are clinically targetable mutations, and two first-generation NTRK tyrosine kinase inhibitors (TKIs), larotrectinib and entrectinib, have been approved by the FDA for the treatment of both adult and pediatric cancers (Figure 1). Second or next-generation NTRK inhibitors, which could overcome the resistance to first-generation NTRK TKIs, are also in clinical trials, including LOXO-195 and TPX-0005 [88,90]. 

Another study documented the impact of NTRK fusions on outcomes in patients with locally advanced or metastatic solid cancers. The NTRK fusion-positive cohort (which included 10 different cancer types with the highest number of patients having CRC) showed a hazard ratio of 1.6 (*p* = 0.05) and a median OS of 10.2 months compared to the NTRK fusion-negative cohort (median OS = 10.4 months). This study suggested that NTRK fusions might be a negative prognostic factor; however, further studies are warranted [91]. 

## 3. Promising Biomarkers

### 3.1. PD-L1

PD-L1 is expressed at low levels on antigen-presenting cells and a variety of nonhematopoietic cells, binding PD-1 on T cells [92]. This interaction inhibits T cell activation and proliferation, suppressing the immune response against tumor cells and allowing them to evade immune surveillance [93]. In theory, higher PD-L1 expression in tumor tissue should confer an improved response to ICI treatment. While PD-L1 is a predictive biomarker in NSCLC, melanoma, and renal cell cancer, its use in CRC is limited [94,95]. PD-L1 expression is induced by IFN-y and TNF-α, secreted by TILs, particularly CD8+ T cells [92]. Therefore, PD-L1 expression by IHC is highly dependent on spatial heterogeneity and sampling. Additionally, the plethora of primary antibodies, staining conditions, and intertumoral heterogeneity add to the technical and biological discrepancy in PD-L1 expression, limiting its use as a biomarker [96]. Analysis of nivolumab plus ipilimumab–treated MSI-H/dMMR CRC showed no difference in ORR utilizing PD-L1 ≥ 1% as a cutoff [97]. Meta-analyses have demonstrated positive tumor cell PD-L1 expression to confer worse OS, but other studies did not find this association or found the opposite effect [98,99,100]. Some studies have highlighted an increased PD-1 expression in immune cells and TILs to confer an improved OS in CRC [100,101]. While the utility of PD-1/PD-L1 as a biomarker requires further research, additional therapies targeting PD-1, such as balstilimab, are being investigated in colorectal cancer (NCT05608044). 

### 3.2. PI3K

*PIK3CA* is a key driver gene that encodes a subunit of phosphatidylinositol 3-kinase (PI3K), a critical component of the PI3K/AKT/mTOR pathway involved in proliferation, survival, and angiogenesis [102]. *PIK3CA* mutations activate the PI3K/AKT/mTOR pathway downstream of RAS activation, conferring resistance to anti-EGFR therapy in *KRAS*-wildtype mCRC, with a more pronounced effect in exon 20 alterations [103,104,105]. The prognostic implication of *PIK3CA* mutations remains unclear in CRC. Although some studies have reported a worse prognostic effect with *PIK3CA* mutations in mCRC, others have reported a null association [104,106]. Evaluation of serabelisib (PIK3 inhibitor) in solid tumor patients with *PIK3CA* mutations is in a phase 1b trial (NCT05300048) (Table 1). 

### 3.3. CXCR4 Axis

CXC chemokine receptor 4 (CXCR4) and its ligand CXCL12 participate in CRC growth, invasion/metastasis, and angiogenesis [107]. Activation of CXCR4 on tumor cells stimulates ICAM-1, enhancing their adhesion to endothelial cells, while CXCR4 on immune cells suppresses the intratumoral immune reaction [108,109,110]. An increased *CXCR4* expression has been linked with an increased liver metastasis and a decreased OS (median 10 months vs. 27 months) in mCRC [111,112]. Additionally, CXCR4 predicts worse recurrence-free survival following curative hepatectomy in CRC patients [113]. Preclinical models have implicated CXCR4 in resistance to oxaliplatin (OXA) and 5-FU [114]. Plerixafor, a small molecular antagonist of the CXCR4 receptor, is being evaluated in phase 1 studies for pancreatic and colorectal cancer (NCT02179970) (Table 1).

### 3.4. CCR5 Axis

The C-C motif chemokine receptor 5 (CCR5)/C-C motif chemokine ligand 5 (CCL5) axis has been implicated in an autocrine and paracrine fashion to stimulate cancer cell proliferation, metastasis, and immune regulation [115]. CCR5 induces vascular endothelial growth factor A (VEGF-A) expression to promote angiogenesis and recruits immunosuppressive tumor-associated macrophages (TAMs) and myeloid-derived suppressor cells (MDSCs) [116]. An increased *CCR5* expression showed a favorable treatment interaction toward bevacizumab, while a decreased expression predicted a response to cetuximab in the CALGB/SWOG 80405 cohort [117]. Additionally, single nucleotide polymorphisms (SNPs) in *CCL5*/*CCR5* have been predictive of a response to cetuximab and regorafenib therapy [118,119,120]. CCR5 may have a prognostic effect, as one study noted that an increased *CCR5* expression displayed worse disease-specific survival, and the CALGB/SWOG 80405 cohort similarly displayed a decreased OS in cetuximab/FOLFOX-treated mCRC [117,121]. Maraviroc, an FDA-approved HIV medication that inhibits *CCR5* expression, showed initial promise in treating refractory mCRC (phase 1 MARACON trial NCT01736813). Given the immunosuppressive role of CCR5 and their expression on immune cells, maraviroc and another CCR5 inhibitor, vicriviroc, are being evaluated in combination with pembrolizumab for MSS mCRC (NCT03274804, NCT03631407) (Table 1).

### 3.5. TIM3/LAG3

T cell immunoglobulin and mucin domain-containing protein 3 (TIM3) and lymphocyte-activation gene 3 (LAG3) are immune checkpoint receptors expressed on T cells, macrophages, and other immune cells, which suppresses antitumor immune responses [122,123]. Within CRC TME, TIM3 and LAG3 promote immune tolerance by inhibiting T cell activity and favoring M2-like polarization of macrophages [124,125]. In stage I-III CRC, an increased tumor *TIM3* and *LAG3* expression correlated with a decreased OS, while an increased expression on immune cells had the opposite effect [126]. Murine models revealed that the co-blockade of TIM3 and its ligand (CEACAM1) leads to enhanced antitumor immune responses, improving CRC clearance [127]. Clinical trials are evaluating TSR-022 (anti TIM3 antibody) in solid tumors, including colorectal cancer (phase 1 NCT02817633). Mouse models demonstrated that combining LAG3 and PD1 inhibitors overcame tumor resistance to either agent alone [128]. Similarly, the evaluation of nivolumab with relatlimab (LAG3 monoclonal antibody) in MSS colorectal cancer patients is underway (phase 2 NCT03642067) (Table 1). After a combination of favezelimab (Anti-LAG3) and pembrolizumab demonstrated safety and robust efficacy in the PD-L1 combined positive score (CPS) ≥ 1 MSS mCRC, a phase 3 trial is comparing it to SOC in PD-L1 positive CRC (NCT05064059) (Table 1) [129].

### 3.6. CEA/CEACAM5

Carcinoembryonic antigen (CEA), a glycoprotein and tumor marker encoded by CEACAM5, is elevated in patients with CRC. Preoperative serum CEA (s-CEA) of > 5 ng/mL confer worse OS and cancer-specific survival [127,128]. In addition to s-CEA, tissue CEA (t-CEA) can be assessed using IHC with expression patterns described as apicoluminal (AL) or diffuse-cytoplasmic (DC). DC pattern t-CEA is associated with a higher rate of recurrence along with a lower OS and disease-free survival (DFS) relative to the AL pattern [129,130,131]. Additionally, increased high-intensity t-CEA is associated with a decreased DFS [131]. The elevated expression of CEACAM5 in colorectal cancer relative to normal tissue highlights its utility as an antibody-drug conjugate (ADC) target. Early data for M9140 (Anti-CEACAM5 ADC with a topoisomerase 1 inhibitor) demonstrated a suitable safety profile and disease control rate (52.5%) in heavily pretreated (≥2 prior lines of therapy) mCRC (NCT05464030) (Table 1) [132]. Cibisatamab (CEACAM5/CD3 T cell–bispecific antibody) is being tested with atezolizumab in mCRC (NCT03866239) (Table 1).

### 3.7. c-MET

Mesenchymal–epithelial transition factor (c-MET) is a receptor tyrosine kinase encoded by *MET*, responsible for cellular proliferation, angiogenesis, and epithelial–mesenchymal transition (EMT) [130]. Several studies, along with a meta-analysis, noted that an increased c-MET expression predicts worse OS and DFS [131,132]. *MET* amplification correlates with acquired resistance to anti-EGFR therapy in CRC without *KRAS* mutations [133]. Combining cetuximab with MET inhibitors suppressed *MET*-induced anti-EGFR resistance in CRC cell lines [134]. Early data on ABBV-400, a c-MET-targeted ADC linked with telisotuzumab (topoisomerase 1 inhibitor), demonstrated a robust safety profile and ORR (61.9%) in CRC (NCT05029882, NCT06107413) (Table 1) [135].

### 3.8. ARID1A

ARID1A is a subunit of the SWI/SNF complex, which plays a role in regulating DNA repair. A loss of function of the SWI/SNF complex promotes genomic instability and tumor progression. In CRC, ARID1A is a known driver gene, and mutations are associated with right-sided tumors, MSI-H/dMMR, and BRAF mutations [136]. Studies suggest that ARID1A mutations may be implicated in both intrinsic and acquired resistance to cetuximab [137]. Patients enrolled in CALGB/SWOG 80405, a randomized phase III study evaluating the efficacy of chemotherapy plus cetuximab vs. chemotherapy plus bevacizumab in first-line CRC, who had *ARID1A* mutations at baseline, had poorer clinical outcomes with cetuximab compared to bevacizumab. These findings were supported in patient-derived xenograft models of extended RAS/*BRAF* wildtype tumors. In post-treatment cell-free DNA (cfDNA) analysis of these patients, *KRAS* and *ARID1A* mutations were enriched in those who were treated with cetuximab but not bevacizumab. Additional CRC genomic analysis suggests that *ARID1A* and EGFR-pathway mutations are mutually exclusive, consistent with findings in lung cancer. Further exploration is warranted to delineate if *ARID1A* mutations identify a group of patients who benefit from MAPK inhibition, in addition to being a predictive biomarker of a poor response to cetuximab treatment. Phase 2 trials are evaluating tislelizumab (PD-1 Antibody) plus fruquintinib (VEGFR 1/2/3 Inhibitor) for *ARID1A*-mutated MSS mCRC (NCT05690035) (Table 1).

### 3.9. PLK1

Polo-like Kinase 1 (PLK1) is a serine/threonine protein kinase family that plays a role in cell cycle progression by regulating progression to mitosis at the G2/M checkpoint [138]. It also plays a role in DNA damage response and cell death pathways. PLK1 is overexpressed in many tumor types and associated with poor prognosis, making it a promising therapeutic target. Higher levels of PLK-1/phosphorylated-PLK1 were found in relapsed/mCRC tissues compared to matched primary CRC tissues, suggesting that it is a marker of poor prognosis and resistance to oxaliplatin-based chemotherapy [139]. Inhibition of PLK1 in CRC cell lines demonstrated that PLK1 inhibitors may attenuate oxaliplatin resistance [139]. A Phase 1b/2 trial studied onvansertib, an oral highly selective PLK1 inhibitor, in combination with FOLFIRI and bevacizumab as a second-line treatment for *KRAS*-mutated mCRC and found that the combination was well-tolerated with durable responses [140]. A Phase 2 trial is underway to assess its utility combined with chemotherapy in the first-line setting for *KRAS-* or RAS-mutated mCRC (NCT06106308) (Table 1).

## 4. The Role of Liquid Biopsy/ctDNA

Liquid biopsies (circulating tumor DNA [ctDNA]) are valuable tools in the management of CRC. When a tumor cell dies, or through active secretion, ctDNA is released in body fluids, most commonly blood [141]. This allows for the detection of mutations, chromosomal rearrangements, methylation patterns, and different fragment lengths [142]. Liquid biopsies can be performed serially during a patient’s disease course and used to interpret tumor biology and assess tumor response to therapy [143]. Although tissue biopsy is the gold standard for molecular profiling, it has several limitations, including insufficient tissue sampling and a longer turnaround time. A prospective study from Japan noted ctDNA to have a lower sequencing failure rate (0.1% vs. 10.6%) and a shorter screening time (11 vs. 33 days, sample acquisition + test duration), ultimately leading to a higher clinical trial enrollment (9.5% vs. 4.1%) [144]. Tumor heterogeneity and evolution have necessitated resampling with liquid biopsy mutations that confer resistance to treatments such as *RAS* mutations in those receiving anti-EGFR therapy [145]. The concordance of tissue and liquid biopsy is acceptable, ranging from 77–96%, with NGS-based methods [145].

ctDNA has shown significant promise in detecting minimal residual disease (MRD) and monitoring for early recurrence in CRC. A prospective study of postsurgical stage III CRC highlighted that MRD positivity following adjuvant chemotherapy is significantly associated with an increased risk of recurrence (HR, 7.5; 95% CI, 3.5–16.1; *p* < 0.001) [146]. These findings were replicated in several studies, even demonstrating prognostic value with ctDNA positivity having worse OS [147,148]. The prognostic utility of ctDNA has been leveraged to select CRC patients likely to benefit from adjuvant chemotherapy (ACT), reducing unnecessary toxicities. Stage II/III CRC patients were prospectively randomized to SOC or a ctDNA-guided protocol for ACT and demonstrated noninferior DFS and OS [149]. When evaluating metastatic gastrointestinal cancers, a four week decrease following systemic therapy initiation predicted the clinical benefit and PFS [149]. Several trials are utilizing dynamic changes in ctDNA to guide treatment decisions in mCRC (NCT04786600, NCT03844620, NCT05062317). Liquid biopsy lacks sensitivity in detecting fusions and copy number losses while having a higher TMB relative to tissue biopsy [150]. The utility of ctDNA has broadened from its initial use in the advanced/metastatic setting for molecular profiling and detection of acquired resistance mechanisms to the identification of MRD and early detection.

## 5. Future Directions

NGS has altered the paradigm of genomics research, offering unparalleled capabilities for analyzing DNA and RNA molecules in a high-throughput and cost-effective manner. Third-generation sequencing has allowed for longer read lengths, real-time sequencing, minimal sample preparation, and direct detection of epigenetic modifications, providing a comprehensive understanding of genomic structure [151]. Using NGS in ctDNA offers a minimally invasive approach to monitor disease progression and treatment response. Studying the dynamics of these biomarkers over time and across different treatment regimens can provide valuable insights into the tumor landscape and inform personalized treatment strategies [152].

CRC is known to exhibit a high intratumor heterogeneity, with different subpopulations of tumor cells harboring distinct genetic and transcriptomic profiles. Single-cell RNA sequencing (scRNA-seq) evaluates the heterogeneity at the single-cell level, enabling the identification of rare cell subpopulations that may play crucial roles in disease progression, metastasis, and treatment resistance [153]. Additionally, scRNA-seq can delineate the complex interactions between tumor cells and immune cells, potentially identifying biomarkers related to immunosuppression or immune evasion.

Multiomics integrates genomic, transcriptomic, proteomic, and epigenomic data along with clinical metadata through artificial intelligence (AI) and bioinformatics tools. By combining data from various omic levels, researchers can gain a more comprehensive understanding of the underlying mechanisms, regulatory pathways, and functional relationships with CRC. Utilizing feature selection and extraction techniques such as principal component analysis, researchers can reduce the dimensionality of multiomics data while preserving the variance and information [154]. Machine learning (ML) models have demonstrated a remarkable accuracy in predicting responses to first-line chemotherapy based on multiomics data [155].

With advances in molecular testing and modeling, we highlight emerging evidence on novel pathways and biomarkers that may double as therapeutic targets. HLAs are divided into two classes, HLA class I and class II, which present peptides to CD8+ T cells and CD4+ T cells, respectively. Reduced expression of HLA class I genes has been associated with immune evasion in immunotherapy-naive MSI-H/dMMR CRC, suggesting a possible explanation for primary resistance to ICI [156,157]. HLA class I and II SNPs predict outcomes in first-line treatment in mCRC, with differential effects based on biologic agent and chemotherapy backbone [158,159]. While not prognostic, MCL-1, an anti-apoptotic protein from the BCL-2 family, induces chemoresistance and plays a crucial role in CRC by maintaining intestinal homeostasis [160,161,162]. Given that MCL-1 and BCL-xL are co-expressed in late-stage CRC, this chemoresistance can be countered by coadministration of A-1331852 (BCL-xL inhibitor) and A-1210477 (MCL-1 inhibitor) [162]. A high MCL-1 expression also correlates with an increased M1 and M2 macrophage infiltration in CRC tumors, affecting cancer cell proliferation and EMT [163,164].

The circadian rhythm, regulated by the core clock genes (*BMAL1* and *CLOCK*), is another pathway closely linked with CRC proliferation and metastasis [165,166]. BMAL1 suppresses a hippo-dependent, self-renewal pathway in the intestinal epithelium such that the loss of *BMAL1* in knockout mouse models results in the upregulation of stem cell signaling and increased CRC growth [167]. *BMAL1* drives the transcription of *VEGFA* conferring resistance to bevacizumab treatment, therefore combining bevacizumab with a *CRY2* stabilizer (SHP1705) to inhibit BMAL1 activity led to a decreased tumor volume and an improved OS in mouse models [168]. Like BMAL1, dihydropyrimidine dehydrogenase (DPD) is another protein involved in therapeutic resistance. A high tumoral DPD expression is associated with 5-FU resistance in CRC; consequently, combining 5-FU with DPD inhibitors (S1PR2 inhibitors or SLR080811) improves efficacy [169,170,171]. DPD expression positively correlates with CD4+ T cells, CD8+ T cells, and macrophages in the TME, in addition to the expression of immune checkpoint markers (PD-L1 and PD-1) [172]. This suggests that DPD not only has a predictive value for 5-FU resistance but also holds potential for immunotherapy-treated CRC.

Both neurotransmitters and ferroptosis play a role in modulating the immune response and are growing areas of CRC research. Neurotransmitters in gastrointestinal tumors facilitate interactions between cancer and immune cells, promoting oncogenic processes [173,174,175,176]. The expression of neurotransmitter receptors on immune cells affects tumor immune responses and may influence immune-targeting treatments [174]. Blocking receptors like muscarinic receptor 3 (M3R) and monoamine oxidase A (MAOA) have shown decreased tumor growth and altered immune responses in CRC models [177,178]. These findings suggest that neurotransmitters have potential as predictive biomarkers and may pave the way for novel therapies targeting neurotransmitters to overcome therapy resistance [179]. Ferroptosis, an iron-dependent form of cell death, is regulated by genes involved in iron metabolism and has been implicated in CRC tumor biology [180,181,182]. In CRC, the altered expression of ferroptosis-related genes predicts OS and the immune response [183,184,185,186,187]. Ferroptosis is being explored in combination therapy with ICIs as a promising treatment strategy to increase sensitivity and overcome resistance [188,189]. Glutamine metabolism regulates ferroptosis by contributing to the synthesis of glutathione (GSH) and through the process of glutaminolysis [190]. Oncogenic KRAS reprograms cellular metabolism, limiting these processes and thereby making KRAS-mutant tumors more susceptible to ferroptosis induction as a potential clinical strategy [191].

As the era of targeted therapies continues to evolve, the development of companion diagnostics and predictive biomarkers becomes increasingly crucial. Identifying biomarkers that can accurately predict the response to targeted agents will maximize therapeutic efficacy while minimizing unnecessary toxicities and healthcare costs.

## 6. Conclusions

The predictive capacity of MSI-H, RAS, and *BRAF* has necessitated a molecular-targeted approach in CRC. The MSI status confers substantial improvements in clinical outcomes and decreased toxicities through ICI, offering patients long-term survival or near curative results [69]. *BRAF* V600E had a negative prognostic value but is now predictive of the response to combined targeted BRAF/EGFR inhibition, doubling survival over standard treatments [51]. While the prevalence of these alterations constitutes the minority of CRCs, they underscore the necessity of identifying predictive biomarkers.

## Figures and Tables

**Table 1:** Clinical trials evaluating therapeutic potential in KRAS mutations and promising biomarkers.

Gene	Molecular Criteria	Setting	Therapy of Interest	Trial Number/Phase	Status
KRAS	KRAS G12C mutation	mCRC	Sotorasib (G12C inhibitor) + panitumumab	NCT05198934 (Phase 3)	Active
		mCRC	Adagrasib (G12C inhibitor) + cetuximab	NCT03785249 (Phase 1/2)	Active
		mCRC	Adagrasib (G12C inhibitor) + cetuximab	NCT04793958 (Phase 3)	Active
		mCRC	Sotorasib (G12C inhibitor) + panitumumab + FOLFIRI	NCT06252649 (Phase 3)	Not yet recruiting
		mCRC	Divarasib (G12C inhibitor) + cetuximab	NCT04449874 (Phase 1)	Active
		Advanced solid tumors	Glecirasib (G12C inhibitor) + JAB-3312 (SHP2 inhibitor)	NCT05288205 (Phase 1/2)	Active
		Advanced solid tumors	Opnurasib (G12C inhibitor)	NCT04699188 (Phase 1/2)	Active
		Advanced solid tumors	Divarasib (G12C inhibitor)	NCT04449874 (Phase 1)	Active
	KRAS mutated (except G13 mutations)	mCRC	Vociprotafib (SHP2 inhibitor) + Temuterkib (ERK ½ inhibitor)	NCT04916236 (Phase 1)	Active
	KRAS G12D mutation	mCRC	Adagrasib (G12C inhibitor) + Batoprotafib (SHP2 inhibitor)	NCT04330664 (Phase 1/2)	Active
		Advanced solid tumors	MRTX1133 (G12D inhibitor)	NCT05737706 (Phase 1/2)	Active
		Advanced solid tumors	RMC-9805 (G12D inhibitor)	NCT06040541 (Phase 1)	Active
	KRAS G12V mutation	Advanced solid tumors	AFNT-211 (G12V TCR)	NCT06105021 (Phase 1/2)	Active
		Advanced solid tumors	FHA11 (G12V TCR)	NCT06043713 (Phase 1)	Active
	KRAS G12D, G12V, G13D or G12C	Non-MSI-H/dMMR mCRC	mRNA-5671/V941	NCT03948763 (Phase 1)	Completed (2022)
	RAS mutation	Advanced solid tumors	RMC-6236 (RAS^multi^ inhibitor)	NCT05379985 (Phase 1)	Active
CEACAM5		Advanced solid tumors	M9140 (CEACAM5 ADC)	NCT05464030 (Phase 1)	Active
	High CEACAM5 Expression	Previously treated MSS mCRC	Obinutuzumab (CD20 mAb) + Cibisatamab (CEA-CD3 bispecific Ab) + atezolizumab (PDL1 mAb)	NCT03866239 (Phase 1)	Completed (2024)
c-MET		Advanced solid tumors	ABBV-400 (MET Ab) + Bevacizumab	NCT05029882 (Phase 1)	Active
		Previously treated unresectable mCRC	ABBV-400 (MET Ab) + 5FU + Folinic acid + Bevacizumab	NCT06107413 (Phase 2)	Active
PIK3CA	PIK3CA mutation	Advanced solid cancers including CRC	Serabelisib (PI3Kα inhibitor)	NCT05300048 (Phase 1b)	Active
	PIK3CA mutation	mCRC	Alpelisib (PI3Kα inhibitor) + capecitabine	NCT04753203 (Phase 1b/2)	Active
CXCR4		Advanced pancreatic, ovarian, and CRC	Plerixafor (CXCR4 inhibitor)	NCT02179970 (Phase 1)	Completed 2018
CCR5		mCRC	Maraviroc (CCR5 inhibitor)	NCT01736813 (phase 1)	Completed 2014
		Advanced solid cancers including CRC	OB-002 (CCR5 antagonist)	NCT05940844 (Phase 1)	Not yet recruiting
	MSS	mCRC	Vicriviroc (CCR5 inhibitor) + pembrolizumab	NCT03631407 (Phase 2)	Completed 2021
		Advanced pancreatic and CRC	Ipilimumab + Maraviroc (CCR5 inhibitor) + nivolumab	NCT04721301 (Phase 1)	Completed 2023
	MSS	mCRC	Maraviroc (CCR5 inhibitor) + pembrolizumab	NCT03274804 (Phase 1)	Completed 2020
TIM3	None for inclusion but evaluating TIM3 expression levels	Advanced solid cancers including CRC	TSR-022 (anti-TIM3) + multiple ICI/chemotherapy arms	NCT02817633 (Phase 1)	Active
LAG3		Advanced solid cancers including CRC	TSR-033 (LAG3 antibody) + dostarlimab	NCT03250832 (Phase 1)	Completed 2023
	MSI-H	Localized and locally advanced CRC	Fianlimab (LAG3 inhibitor) + Cemiplimab	NCT06205836 (Phase 1)	Active
	MSS, cohort A CPM ≥ 15%, cohort B CPM < 15%.	Metastatic or locally advanced CRC	Relatlimab (LAG3 antibody) + Nivolumab	NCT03642067 (Phase 2)	Active
		Advanced solid cancers including CRC	XmAb^®^22841 (CTLA-4 × LAG3 bispecific antibody) + Pembrolizumab	NCT03849469 (Phase 1)	Completed 2023
	MSS CPS ≥ 1	mCRC	Favezelimab (LAG3 antibody) + Pembrolizumab	NCT05064059 (Phase 3)	Active
ARID1A	MSS and ARID1A mutation	mCRC	Tislelizumab (PD-1 Antibody) + Fruquintinib (VEGFR 1/2/3 Inhibitor)	NCT05690035 (Phase 2)	Active
PLK1	KRAS mutated and BRAFV600E negative	mCRC	Onvansertib (PLK1 inhibitor) + FOLFIRI + Bevacizumab	NCT03829410 (Phase 1b/2)	Completed 2024
	KRAS mutated and BRAFV600E negative	mCRC	Onvansertib (PLK1 inhibitor) + Bevacizumab + FOLFIRI or FOLFOX	NCT06106308 (Phase 2)	Active
	KRAS or NRAS Mutation	mCRC	Onvansertib (PLK1 inhibitor) + Bevacizumab + FOLFIRI	NCT05593328 (Phase 2)	Active

CRC = colorectal cancer; mCRC = metastatic colorectal cancer; MSS = microsatellite stable; CPM = composite PD-L1/Mucin; CPS = combined positive score.

## Abbreviations

5-FU	5-fluorouracil
ADC	antibody-drug conjugate
AI	artificial intelligence
AL	apicoluminal
BRAFV600E	BRAF mutation with valine substitution for glutamic acid at amino acid 600
c-MET	mesenchymal–epithelial transition factor
CCR5	C-C motif chemokine receptor 5
CEA	carcinoembryonic antigen
cfDNA	cell-free DNA
CMS	consensus molecular subtype
CRC	colorectal cancer
ctDNA	circulating tumor DNA
CXCR4	CXC chemokine receptor 4
DC	diffuse-cytoplasmic
DFS	disease-free survival
DPD	dihydropyrimidine dehydrogenase
EGFR	epidermal growth factor receptor
EMT	epithelia–mesenchymal transition
FDA	Food and Drug Administration
FISH	fluorescence in situ hybridization
GSH	glutathione
HLA	human leukocyte antigen
ICI	immune checkpoint inhibitor
IHC	immunohistochemistry
LAG3	lymphocyte-activation gene 3
M3R	muscarinic receptor 3
MAOA	monoamine oxidase A
Mb	megabase
mCRC	metastatic colorectal cancer
MHC	major histocompatibility complex
ML	machine learning
MRD	minimal residual disease
MSI-H/dMMR	microsatellite instability or mismatch repair deficiency
MSS	microsatellite stable
NCCN	National Comprehensive Cancer Network
NGS	next-generation sequencing
NR	not reached
NSCLC	non-small cell lung cancer
OS	overall survival
PFS	progression-free survival
PI3K	phosphatidylinositol 3-kinase
PLK1	polo-like Kinase 1
s-CEA	serum carcinoembryonic antigen
scRNA-seq	single-cell RNA sequencing
SNP	single nucleotide polymorphism
SOC	standard of care
t-CEA	tissue carcinoembryonic antigen
TAMs	tumor-associated macrophages
TCR	T cell receptor
TILs	tumor infiltrating lymphocytes
TIM3	T cell immunoglobulin and mucin domain-containing protein 3
TKI	tyrosine kinase inhibitor
TMB	tumor mutational burden
TME	tumor microenvironment
Treg	regulatory T cell
VEGF-A	vascular endothelial growth factor A
WES	whole exome sequencing
